# Supplementary material for: Learning curve and surgical outcome of robotic assisted colorectal surgery with ERAS program
Source: Sci Rep. 2022 Nov 29;12:20566. doi: 10.1038/s41598-022-24665-w (PMC9709162; doi:10.1038/s41598-022-24665-w)
Supplement: Supplementary file 2 — Supplementary Information 2. [file 41598_2022_24665_MOESM2_ESM.zip › RAL Raw data-Ñ[▒K/084/OP N_ñw╝╨░O▒Kñσ.pdf]

W92 / W92

臺中榮民總醫院
手術室護理記錄

[redacted]

基本資料

- 室別： 開刀房-17室，手術科別： CRS，手術類別： 常規，日期： 2021/05/15
- 進入時間： 0750 ； 麻醉開始時間： 0800 ； Time Out時間： 0955 ； 劃刀時間： 0956 ； 手術結束時間： 1410 送出時間： 1430 ；
- 麻醉方式： GE
  診斷：Malignant tumor of sigmoid colon
  手術名稱：Robot Assisted Low Anterior Resection
  術前預防性抗生素：
  - 抗生素名稱 ： Veterin 1GM IVD由麻醉護理師執行
  - 時間： 0930
  - 上次簽章: 詹佳穎

  ，追加預防性抗生素：
- 手術醫師： 蔣鋒帆，林陳彥，羅于珍
- 麻醉醫師： VS 蔡志忠
-
  - 刷手護士A ： 邱玟琇，時間： 0750，刷手護士B ： 蘇敬涵，時間： 0750
  - 流動護士A ： 詹佳穎，時間： 0750

皮膚狀況

A-V Shunt： 無
術前： 完整
記錄者： 上次簽章: 詹佳穎
術後： 完整
記錄者： 上次簽章: 詹佳穎

手術準備

手術臥式 單一手術臥式

-
  - 手術臥式： 截石臥式
  - 使用附件： 溫毯 39 ℃，腳架
  - 皮膚準備： 10%Tr.Betadine，10%Aq Betadine，Hibiscrub
  - 電燒： 使用電燒: 電極片位置： 右大腿，記錄者： 上次簽章: 詹佳穎

止血帶T： 無

植入物

無

標本送檢

- 細菌培養 無
- 細胞學 無
- 冰凍切片 無
- 病理標本
  組織名稱一：IMA root
  ,
  組織名稱二：rectum
  ,
  組織名稱三：distal cut end
- 截肢 無

標本交付 標本： 2 袋，標本： 0 盒，標本： 1 瓶，細胞學： 0 管，Culture Tube： 0 管，被交付醫師： CR 林陳彥，

- 記錄者： 上次簽章: 詹佳穎
- 時間： 1300

傷口術後敷料

傷口敷料： 傷口敷料： 無

- 請於圖上標示部位：

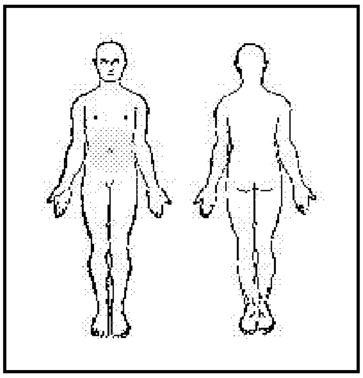

- 傷口敷料： 紗布

導管

導管	天數	導管資訊	插入日期	插入時間	更換日期	拔除日期	拔除時間	拔除原因	預計拔除/更換	預計拔除/更換日期
A-line (1)	1	號碼:20 G; 部位:右手;	2021/05/15	0815						
Foley(1)	1	號碼:16 Fr.; 部位:膀胱;	2021/05/15	0840						
Double Lumen CVC(1)	1	號碼:8 Fr; 部位:jugular (右); 象限:RU;	2021/05/15	0835						
Epidural PCA(1)	1	部位:硬脊膜下;	2021/05/15	0810						

用物計數

- 手術中出血量： minimal c.c.
- 手術中尿量： 500 c.c.
-
    - 其它： 無

用物計數： 多切口同時縫合

- 1.器械： 正確，2.縫針： 正確，3.刀片： 正確，4.紗布： 正確，5.腹部墊： 未使用，6.紗球： 未使用，7.PEANUTS ： 未使用，8.紗條： 未使用，9.絲帶： 未使用，10.針頭： 未使用，11.Vessel loop： 未使用，12.Boots： 未使用，13.Jelco： 未使用，14.棉條： 未使用，
    - 第一次：
        - 刷手護士 ： 蘇敬涵
        - 流動護士： 上次簽章: 詹佳穎
        - 時間： 1310

  ,
    - 第二次：
        - 刷手護士 ： 蘇敬涵
        - 流動護士： 上次簽章: 詹佳穎
        - 時間： 1315

  ,
    - 第三次：
        - 刷手護士 ： 蘇敬涵
        - 流動護士： 上次簽章: 詹佳穎
        - 時間： 1320

備註

備註： NIL

- 記錄者： 上次簽章: 詹佳穎
- 時間： ０９３０

填寫護理人員 詹佳穎

正式摘要

	W92 / W92

doctorRank:B
